# Gender differences in sociodemographic and behavioural factors associated with BMI in an adult population in rural Burkina Faso – an AWI-Gen sub-study

**DOI:** 10.1080/16549716.2018.1527557

**Published:** 2018-10-12

**Authors:** Romuald Palwende Boua, Hermann Sorgho, Toussaint Rouamba, Seydou Nakanabo Diallo, Joel D. Bognini, Sophie Z. Konkobo, Daniel Valia, Moussa Lingani, Serge Ouoba, Alain S. Tougma, Biebo Bihoun, Nigel J. Crowther, Shane A. Norris, Michèle Ramsay, Halidou Tinto

**Affiliations:** a Institut de Recherche en Sciences de la Santé, Clinical Research Unit of Nanoro (IRSS/CRUN), Nanoro, Burkina Faso; b Sydney Brenner Institute of Molecular Bioscience (SBIMB), Faculty of Health Sciences, University of the Witwatersrand, Johannesburg, South Africa; c Division of Human Genetics, National Health Laboratory Service and School of Pathology, Faculty of Health Sciences, University of the Witwatersrand, Johannesburg, South Africa; d Centre de Recherche en Epidémiologie, Biostatistique et Recherche Clinique, Ecole de Santé Publique, Université libre de Bruxelles, Brussels, Belgium; e Department of Chemical Pathology, National Health Laboratory Service, Faculty of Health Sciences, University of the Witwatersrand, Johannesburg, South Africa; f MRC/Wits Developmental Pathways for Health Research Unit (DPHRU), University of the Witwatersrand, Johannesburg, South Africa

**Keywords:** BMI distribution across African communities, BMI, underweight, problematic alcohol consumption, chewing tobacco, hierarchical model, structural equation model

## Abstract

**Background**: The global health transition is linked with an increased burden of non-communicable diseases with cardiovascular diseases leading the epidemic. In sub-Saharan Africa (SSA), the prevalence of obesity has increased during the past decades and there is a need to investigate the associated driving factors. In Burkina Faso obesity remains low, especially in rural areas. In this study we recruited middle-aged adults, as part of a larger study on genetic and environmental contributions to cardiometabolic disease among Africans.

**Objectives**: To investigate the distribution of BMI and prevalence of obesity in a cross-sectional population-based study and to determine the sociodemographic and behavioural correlates with BMI.

**Methods**: Participants (N = 2,076) were recruited from the Nanoro Health and Demographic Surveillance System area and were aged 40–60 years. We applied hierarchical modelling to identify factors associated with BMI and structural equation modelling to identify mediated effects of sociodemographic and behavioural variables on BMI.

**Results**: Data are presented on 2,076 participants (49.9% female). Men had significantly higher BMI than women with medians of 21.1 (19.2 – 23.4) vs 19.8 (18.1 – 21.6) (p < 0.001), and there were significantly more underweight women compared to men (31.0% vs 17.4%) (p < 0.001). More men were overweight and obese than women (11.9% vs 5.2% and 2.2% vs 1.4%). Socioeconomic status was the major contributor to increased BMI for men, and education was the main contributor in women. Tobacco smoking and chewing, and problematic alcohol consumption were associated with a decrease in BMI in men and women.

**Conclusion**: Overweight and obesity are relatively low among adults in rural Burkina Faso, and men had a higher median BMI than women. Behavioural factors, including tobacco use and alcohol consumption, contributed to a decrease in BMI, whereas socioeconomic status and education (which were both generally low in this community) contributed to an increase in BMI.

## Background

During the last two decades, the global burden of cardiovascular diseases (CVDs) has increased considerably with low- and middle-income countries (LMIC) now harbouring about 80% of the worldwide burden [1]. Sub-Saharan Africa (SSA) is experiencing a health and demographic transition that has shifted the major causes of death from communicable and nutritional diseases to non-communicable diseases (NCDs). Hence, deaths due to NCDs are projected to increase by 15% globally between 2010 and 2020 and the greatest increases are expected in Africa, the Eastern Mediterranean, and South-East Asia, where they will increase by over 20% [2]. Among the common risk factors for CVDs, body mass index (BMI) is often used to assess a population’s risk because it is easily measured, inexpensive and associated with all-cause mortality [3]. Despite the controversy regarding the use of BMI [4–7] to predict disease, BMI remains the most commonly used marker of whole body obesity. By 2014, it was estimated that approximately a billion adults were overweight and that 600 million were obese worldwide [8]. Within SSA, the prevalence of obesity has been increasing over the past 30 years with regional disparities [9]. Whereas most hypotheses emphasise the role of urbanization and a westernization of lifestyles as the main causes of the rising prevalence of obesity in some parts of SSA [10–12], little reliable data have been collected to explain the trends at national or regional levels.

Burkina Faso is a ‘Low Human Development’ country, with a gross national per capita annual income of USD 1,537 in 2015. It has considerable income inequalities with a Gini index of 39.5 [13] and about 80% of the population is rural. Nanoro is located within the livelihood zone called the ‘central plateau and market gardening’. This Sudano-Sahelian region is characterized by rainfed agriculture (especially for market gardening) and livestock herding, with an annual rainfall of 600–700 mm. Family farming is the main source of income, followed by trade and traditional gold mining [14].

In Burkina Faso, the only country-level data on obesity is in children under five years of age [15] and in women aged 15–49 years, generated from a demographic and health survey (DHS) [16]. In 2013, the Minister of Health (MoH) conducted the first nationwide survey for NCDs in Burkina Faso according to the WHO STEPwise approach to surveillance (STEPS) methodology and reported an overall prevalence of obesity of 2.1% with sex disparities [17]. Although these surveys provided some useful information, they did not provide sufficient data to fully understand the BMI distribution and trends in this region or to define the environmental and behavioural factors that influence BMI.

We investigated BMI in a rural population of Burkina Faso aged 40–60 years, as part of the AWI-Gen study (Africa Wits-INDEPTH partnership for Genomic Research) [18]. The objective of the present study is to describe the distribution of BMI and to identify sociodemographic and behavioural factors associated with BMI in rural Burkina Faso.

## Methods

This is a sub-study of AWI-Gen, a multi-centre study that aims to investigate genomic and environmental risk factors for cardiometabolic diseases in African populations. The AWI-Gen study was implemented at the Health and Demographic Surveillance System (HDSS) area of the Clinical Research Unit of Nanoro (CRUN), Centre-West Direction of Health Research Institute (IRSS/DRCO), in Burkina Faso. The study participants were enrolled from February 2015 to July 2016 after providing informed consent. Inclusion criteria were that they reside in the area of the HDSS, are aged between 40–60 years, not closely related (i.e., first-degree relatives) and women should not be pregnant. It was a cross-sectional population-based study and all study procedures on each individual were performed on the same day. More detail is provided in the AWI-Gen resource paper [19] and data collection procedures are described by Ali et al., (2018, this issue), and briefly outlined below.

### Anthropometry

#### Weight and height measurements

Participants were weighed using a calibrated Seca 813© electronic scale (Seca GmbH Company, Hamburg, Germany). Participants were asked to remove shoes, heavy clothing and jewellery, and to empty their pockets prior to being weighed. Weight was recorded in kg to one decimal place (100g precision) and the coefficient of variation (intra observer variability) was 1.4%. Standing height was measured with participants in their bare feet. Standing height was measured in mm using a Harpenden 602VR© digital stadiometer (Holtain Limited, Crosswell, UK). The coefficient of variation (intra observer variability) was 2.2%. BMI was defined as the weight in kilograms divided by the square of height in meters. When categorized we used cut-offs as follow: underweight: BMI≤ 18.5; normal weight: 18.5< BMI≤ 25; overweight: 25< BMI≤ 30; and obesity: BMI> 30).

### Questionnaire

The questionnaire was administered in the spoken language (Moore, Gourounsi, French, Dioula) of the participants by trained interviewers. Data were collected on demography, family composition, pregnancy and maternity history, matrimonial status, education, employment, household assets, substance use, general health, recent infection history, diet, cardiometabolic risk factors, thyroid and kidney disease, physical activity and sleep. Household assets were used to construct socioeconomic status (SES) quintiles by principal component analysis (PCA) (19). The CAGE (Cut down, Annoyed, Guilty, and Eye opener) questionnaire was used to categorize alcohol consumption into: never consumed; current non-problematic consumer; current problematic consumer; and former consumer. Problematic alcohol use was assigned if the respondent answered ‘yes’ to at least 2 of the 4 questions related to potential problematic alcohol consumption.

### Data analysis

The distributions of continuous variables are reported as median and interquartile range and categorical variables as percentages. To compare data across groups, we used χ^2^ or Fisher’s test, and the Student t-test or Mann-Whitney-Wilcoxon test. As the first step of the hierarchical model we performed bivariate analyses (Fisher F test) to determine correlates with BMI. In accordance with recommendations [20,21], all variables with p < 0.2 in the bivariate analysis were included in a multivariate hierarchical regression analysis with BMI as the dependent variable in all models. This involved building three multivariable linear regression models: Model 1 included sociodemographic variables (age, ethnicity, education level, marital status and SES (quintiles)); Model 2 included lifestyle/behavioural variables (alcohol consumption, tobacco exposure, minutes moderate and vigorous physical activity per week (MVPA) and sedentary behaviour) plus all the significant variables from Model 1; and finally, Model 3 used the significant variables from Model 2 with the addition of menopausal status in the women’s model. We adjusted for tuberculosis (TB) in both groups and by parity in the women’s group.

To better understand the outcome of the hierarchical model, structural equation modelling (SEM) was used to identify direct and indirect effects. This approach represents, estimates and tests a theoretical network of mostly linear relationships between variables that may be either directly observable or observable only through another variable (indirect observation). It is a generalization of both regression and factor analysis and comprises mostly of the linear modelling methods. The procedure places emphasis on covariance structures rather than cases. The fundamental hypothesis in using SEM is that the covariance matrix of the observed variables is a function of a set of parameters. If the model is correct and the parameters are known, then the population covariance matrix would be exactly reproduced by SEM. SEM proceeds by assessing whether a sample covariance or correlation matrix is consistent with a hypothetical matrix implied by the model. The inputs are either raw data or sample moments computed from the data, and a model to be evaluated. The sample moments will include either correlations or variances and covariances. The measurement model defines relations between the observed and unobserved latent variables. The structural model defines relations among the unobserved variables by specifying the pattern by which particular latent variables directly or indirectly influence other latent variables in the model. SEM is mainly a confirmatory technique rather than exploratory and is used to determine whether a certain model is valid, rather than to find a suitable model.

### Software and packages

Statistical analyses were performed using the R software version 3.3.1 (R Development Core Team, R Foundation for Statistical Computing, Vienna, Austria), including the following packages: ‘“sem”’, ‘“lavaan”’ and ‘“semPlot”’.

## Results

### Population characteristics

This paper presents results for 2076 individuals. The population characteristics are presented in Table 1. The median age was the same between men and women and the study population was mainly from the ‘Mossi’ ethnic group (93.2%) with a small proportion of ‘Gourounsi’ (5.3%). The level of education was very low with 83.0% having no formal education, and men were more likely to be educated at all levels than women. Almost everyone in the population was employed (self-employed, formal full-time employment, part-time employment, informal employment), considering that in the rural area self-employment means that they are working on their own farms and cultivating their own lands. The socioeconomic status (SES) was based on household goods, and individuals were divided into wealth quintiles (Q1 to Q5). Significantly more women than men were in the lowest quintile (Q1) and the opposite for the highest quintile (Q5) (p < 0.001).

**Table 1.** Distribution of sociodemographic, behaviour, diet, physical activity, clinical history and anthropometric characteristics by gender.

	Male	Female	P-value	Total
SOCIODEMOGRAPHICS				
Age (years)*	50 (45 – 55)	50 (45 – 54)	0.97†	50 (45 – 55)
Ethnicity			**< 0.001**	
Mossi	1003 (96.3)	931 (89.9)		1934 (93.2)
Gourounsi	16 (1.5)	93 (9.0)		109 (5.3)
Others	22 (2.1)	11 (1.1)		33 (1.6)
Marital status			**< 0.001**	
Never married/cohabiting	14 (1.3)	3 (0.3)		17 (0.8)
Currently married/cohabiting	1017 (97.9)	791 (76.6)		1808 (87.3)
Previously married/cohabiting/partner deceased	8 (0.8)	238 (23.1)		246 (11.9)
Highest level of education			**< 0.001**	
No formal education	755 (72.8)	958 (93.3)		1713 (83.0)
Primary	181 (17.5)	57 (5.6)		238 (11.5)
Secondary	85 (8.2)	10 (1.0)		95 (4.6)
Tertiary	16 (1.5)	2 (0.2)		18 (0.9)
Employment			**0.029**	
No	16 (1.5)	5 (0.5)		21 (1.0)
Yes	1022 (98.5)	1027 (99.5)		2049 (99.0)
Household density*	1.6 (1.2 – 2.0)	1.4 (1 – 1.8)	**< 0.001†**	1.5 (1.1 – 2.0)
Household asset status			**< 0.001**	
Quintile 1	136 (13.1)	205 (19.8)		341 (16.4)
Quintile 2	213 (20.5)	189 (18.3)		402 (19.4)
Quintile 3	192 (18.4)	213 (20.6)		405 (19.5)
Quintile 4	182 (17.5)	200 (19.3)		382 (18.4)
Quintile 5	318 (30.5)	228 (22.0)		546 (26.3)
BEHAVIOUR				
Smoking status			**< 0.001**	
Never smoked	778 (74.7)	1033 (99.8)		1811 (87.2)
Former smoker	121 (11.6)	2 (0.2)		123 (5.9)
Current smoker	142 (13.6)	0 (0.0)		142 (6.8)
Snuff_use			0.73‡	
No	1038 (99.7)	1031 (99.6)		2069 (99.7)
Yes	3 (0.3)	4 (0.4)		7 (0.3)
Chewing tobacco use			**< 0.001**	
No	977 (93.9)	728 (70.3)		1705 (82.1)
Yes	64 (6.1)	307 (29.7)		371 (17.9)
Alcohol consumption			**< 0.001**	
Never consumption	271 (26.1)	277 (26.8)		548 (26.5)
Ever consumption	64 (6.2)	139 (13.5)		203 (9.8)
Current non problematic	543 (52.4)	519 (50.2)		1062 (51.3)
Current problematic	159 (15.3)	98 (9.5)		257 (12.4)
DIET				
Servings bread/day*	0.0 (0.0 – 2.0)	0.0 (0.0 – 1.0)	**< 0.001†**	0.0 (0.0 – 2.0)
Fruit serving/day*	0.0 (0.0 – 1.0)	0.0 (0.0 – 0.0)	**< 0.001†**	0.0 (0.0 – 0.0)
Vegetable serving/day*	5.0 (2.0 – 7.0)	3.0 (0.0 – 6.0)	**< 0.001†**	3.0 (1.0 – 7.0)
Eating out/week*	0.0 (0.0 – 2.0)	0.0 (0.0 – 0.0)	**< 0.001†**	0.0 (0.0 – 0.0)
Sugar sweetened beverage/week	0.0 (0.0 – 0.0)	0.0 (0.0 – 0.0)	**< 0.001†**	0.0 (0.0 – 0.0)
PHYSICAL ACTIVITY				
MVPA(mins/week)	2160 (140 – 3060)	2940 (570 – 3600)	**< 0.001†**	2520 (360 – 3360)
Sitting (min/day)	430 (300 – 660)	480 (360 – 624)	**< 0.001†**	480 (331 – 659)
Sleep (hours/night) *	8.0 (7.0 – 9.0)	8.0 (7.0 – 9.0)	0.34†	8.0 (7.0 – 9.0)
CLINICAL HISTORY				
Menopause				
Pre-Menopause	NA	534 (51.6)	NA	534 (51.6)
Peri Menopause	NA	104 (10.0)	NA	104 (10.0)
Post Menopause	NA	397 (38.4)	NA	397 (38.4)
Number of pregnancies	NA	7.0 (6.0 – 9.0)	NA	7.0 (6.0 – 9.0)
Self-reported diabetes status			**0.004**	
No	1008 (97.2)	1013 (98.3)		2021 (97.7)
Yes	16 (1.5)	2 (0.2)		18 (0.9)
Don’t know	13 (1.2)	16 (1.5)		29 (1.4)
HIV positive			**< 0.001†**	
No	179 (96.3)	62 (93.9)		241 (95.6)
Yes	5 (2.6)	4 (6.1)		9 (3.6)
Don’t know	2 (1.1)	0 (0.0)		2 (0.8)
TB positive			0.008‡	
No	1020 (98.2)	1014 (98.3)		2034 (98.0)
Yes	19 (1.8)	10 (1.0)		35 (1.7)
Don’t know	0 (0.0)	7 (0.7)		7 (0.3)
ANTRHOPOMETRICS				
Height (mm)*	1,733 (1689 – 1782)	1,618 (1580 – 1658)	**< 0.001†**	1,673 (1,614 – 1,738)
Weight (kg)*	63.9 (56.7 – 71.5)	51.8 (46.9 – 57.8)	**< 0.001†**	57.3 (50.4 – 65.9)
BMI (kg/m^2^) *	21.1 (19.2 – 23.4)	19.8 (18.1 – 21.6)	**< 0.001†**	20.4 (18.6 – 22.6)
BMI categories			**< 0.001**	
Underweight	181 (17.4)	321 (31.0)		502 (24.2)
Normal weight	713 (68.5)	647 (62.5)		1,360 (65.5)
Overweight	124 (11.9)	53 (5.1)		177 (8.5)
Obese	23 (2.2)	14 (1.4)		37 (1.8)

Values in brackets are percentage unless stated otherwise

* Median and interquartile range in brackets

† Mann-Whitney-Wilcoxon’s test

‡ Fisher exact test

MVPA, moderate to vigorous intensity physical activity

HIV, Human immunodeficiency virus

TB, tuberculosis

BMI, body mass index

### Behavioural data

Lifestyle/behavioural data showed that men were more likely to smoke tobacco with 11.6% being former smokers and 13.6% current smokers compared to women, with 0.2% and 0.0%, respectively (p < 0.001). However, women used chewing tobacco more often (29.7%) compared to men (6.1%) (p < 0.001). The levels of alcohol consumption were similar for men and women (52.4% for men vs 50.2% for women, current non-problematic consumption), even though more women were former alcohol consumers than men (13.5% vs 6.2%; p < 0.001). The prevalence of problematic consumption was estimated at 15.3% in men and 9.5% (p < 0.001) in women. In terms of diet, men consumed significantly more bread and vegetables than women (p < 0.001) and were eating out more often than women (p < 0.001). In the context of this study, bread consumption should be considered as an indicator of ‘eating out’ instead of an indicator of calorie intake, as is the case in other populations. The moderate to vigorous physical activity data showed that women were significantly (p < 0.001) more active than men (2940 min/week (570–3600) vs 2160 mins/week (140–3,060)), even though men were sitting for less time than women (p < 0.001). Regarding menopausal status, we found that 51.6% of women were pre-menopausal, 10.0% were peri-menopausal and 38.4% were post-menopausal. Self-reported TB was higher in men (1.8%) than women (1.0%; p < 0.001) even though the general level was low.

### BMI data

The men displayed significantly higher height and weight than women. The BMI levels followed the same trend in terms of sex difference in favour of men (p < 0.001) with medians of 21.1 (19.2 – 23.4) vs 19.8 (18.1 – 21.6). When classified into BMI obesity-related categories, the sex differences remain in all categories. There were significantly more women underweight than men (31.0% vs 17.4%), and inversely more men were overweight and obese than women (11.9% vs 5.2% and 2.2% vs 1.4%).

#### Multivariate analysis using hierarchical modelling

The outcome of the bivariate analysis is shown in Table 2 and all variables with p-values lower than 0.20 were selected for inclusion in the hierarchical model. After adjustment of Models 1 and 2, in the combined Model 3, we found that age was inversely associated with BMI in men (−0.09, 95% CI: −0.12; −0.05) but not in women. The men who never married or cohabited had about 2 BMI indices lower than those who were currently married/cohabiting (−2.42, 95% CI: −4.16; −0.69). The level of education was significantly associated with an increase of BMI at secondary and tertiary level of education in men, whereas only at primary and secondary level for women. Problematic drinking was associated with a decreased BMI in men by 0.89 units (95% CI: −1.54; −0.24) compared to those who never consumed alcohol, whereas smoking was found to be associated with a decrease of 2 BMI units (−2.0, 95% CI: −2.59; −1.41) compared to those who never smoked (Table 3). Among women, chewing tobacco was associated with a BMI decrease (−0.79, 95% CI: −1.2; −0.37). Regarding the diet, bread consumption was associated with a BMI increase (0.42, 95% CI: 0.27; 0.57) in women, whereas in men the consumption of vegetables was associated with a slight increase of BMI (0.11, 95% CI: 0.03; 0.16). Our study did not show any conclusive results regarding to association between BMI and physical activity. The hierarchical model explained 26.38% of the variability in BMI among men and 16.42% among women.

**Table 2.** Bivariate analysis of factors associated with BMI for male and female participants in rural Burkina Faso.

	Male		Female	
	Coef CI 95%	P value	Coef CI 95%	P value
Age	−0.12 (−0.16; −0.08)	**< 0.001**	−0.06 (−0.09; −0.02)	**0.001**
Ethnicity (Ref: Mossi)				
Gouroussi	−1.01 (−2.77; 0.74)	0.258	−0.16 (−0.83; 0.52)	0.649
Others	2.13 (0.63; 3.64)	**0.006**	1.62 (−0.25; 3.49)	0.090
Marital status (Ref: Currently married/cohabiting)				
Previously married/cohabiting/partner deceased	−0.23 (−2.71; 2.25)	0.856	−0.72 (−1.18; −0.28)	**0.002**
Never married/cohabiting	−2.75 (−4.63; −0.88)	**0.004**	3.82 (0.27; 7.37)	**0.035**
Education level (Ref: No formal education)				
Primary	0.85 (0.29; 1.4)	**0.003**	1.84 (1.02; 2.66)	**< 0.001**
Secondary	2.98 (2.2; 3.77)	**< 0.001**	7.61 (5.72; 9.51)	**< 0.001**
Tertiary	4.69 (2.98; 6.39)	**< 0.001**	4.22 (0.01; 8.44)	0.050
Household asset status (Ref: Quintile = 1)				
2	0.5 (−0.21; 1.21)	0.167	0.02 (−0.6; 0.63)	0.957
3	0.94 (0.21; 1.67)	**0.011**	0.23 (−0.36; 0.83)	0.444
4	1.15 (0.42; 1.89)	**0.002**	0.43 (−0.18; 1.04)	0.164
5	3.54 (2.87; 4.2)	**< 0.001**	1.34 (0.75; 1.93)	**< 0.001**
Employment (Ref: Yes)				
No	0.33 (−1.49; 2.15)	0.720	0.31 (−2.46; 3.09)	0.825
Household density	0 (−0.02; 0.03)	0.731	0 (−0.02; 0.03)	0.840
Smoking status (Ref: Never smoked)				
Current smoker	−1.97 (−2.6; −1.33)	**< 0.001**	-	-
Former smoker	0.23 (−0.44; 0.91)	0.496	−1.06 (−5.44; 3.32)	0.635
Snuff use (Ref: No)				
Yes	−0.77 (−4.82; 3.28)	0.710	0.84 (−2.26; 3.94)	0.596
Chewing tobacco use (Ref: No)				
Yes	−1.88 (−2.78; −0.98)	**< 0.001**	−1.2 (−1.61; −0.78)	**< 0.001**
Consumption alcohol status (Ref: Never consumption)				
Current non problematic	−1.15 (−1.67; −0.64)	**< 0.001**	−0.24 (−0.7; 0.22)	0.302
Current problematic	−1.74 (−2.43; −1.05)	**< 0.001**	−0.28 (−1.01; 0.44)	0.455
Ever consumption	−1.13 (−2.1; −0.16)	**0.022**	0.39 (−0.26; 1.03)	0.232
Menopause (Ref: Pre-menopause)				
Peri-menopause	-	-	0.54 (−0.12; 1.2)	0.110
Post-menopause	-	-	−0.47 (−0.88; −0.06)	**0.024**
Servings bread/day	0.3 (0.21; 0.39)	**< 0.001**	0.65 (0.52; 0.79)	**< 0.001**
Fruit serving/day	0.23 (0.12; 0.34)	**< 0.001**	0.16 (0.05; 0.26)	**0.004**
Vegetable serving/day	0.26 (0.18; 0.34)	**< 0.001**	0.18 (0.11; 0.25)	**< 0.001**
Physical activity	0 (0; 0)	**0.007**	0 (0; 0)	0.148
Sitting (hours/day)	0 (0; 0)	**< 0.001**	0 (0; 0)	0.995
Eating out per week	0.23 (0.13; 0.32)	**< 0.001**	0.42 (0.17; 0.66)	**0.001**
Sugar sweetened beverage/week	0.36 (0.2; 0.52)	**< 0.001**	1.14 (0.51; 1.78)	**< 0.001**
Sleep (hours/night)	−0.5 (−0.67; −0.33)	**< 0.001**	−0.24 (−0.39; −0.08)	**0.003**
HIV positive (Ref: No)				
Yes	−2.9 (−6.05; 0.25)	0.071	−0.42 (−3.55; 2.71)	0.793
Don’t know	−2.28 (−7.22; 2.65)	0.364	−2.51 (−3.31; −1.72)	**< 0.001**
Not answered	−1.26 (−1.83; −0.69)	**< 0.001**	−0.79 (−2.76; 1.17)	0.429
TB positive (Ref: No)				
Yes	−0.64 (−2.26; 0.98)	0.439	0.61 (−1.74; 2.96)	0.610
Not answered	−2.79 (−7.75; 2.17)	0.270	0.1 (−3; 3.2)	0.951
Parity	-	-	0.02 (−0.05; 0.1)	0.539
Diabetes positive (Ref: No)				
Yes	4.6 (2.8; 6.4)	**< 0.001**	9.8 (5.46; 14.15)	**< 0.001**
Don’t Know	−0.4 (−2.34; 1.53)	0.682	−0.01 (−1.56; 1.53)	0.988

**Table 3.** Hierarchical modelling of factors associated with BMI for males and females in rural Burkina Faso.

	Model 1				Model 2				Model 3			
	Male		Female		Male		Female		Male		Female	
	β coefficient (CI 95%)	P	β coefficient (CI 95%)	P	β coefficient (CI 95%)	P	β coefficient (CI 95%)	P	β coefficient (CI 95%)	P	β coefficient (CI 95%)	P
Age	−0.09 (−0.12; −0.05)	**< 001**	−0.03 (−0.07; 0)	0.067	−0.08 (−0.11; −0.04)	**< 001**	−0.01 (−0.04; 0.03)	0.659	−0.08 (−0.11; −0.04)	**< 001**	0 (−0.04; 0.03)	0.822
Ethnic (Ref: Mossi)												
Gouroussi	−1.32 (−2.94; 0.3)	0.110	−0.16 (−0.82; 0.49)	0.621	−1.28 (−2.86; 0.3)	0.112	−0.12 (−0.76; 0.52)	0.708	−1.27 (−2.85; 0.31)	0.115	−0.11 (−0.76; 0.53)	0.729
Others	0.15 (−1.45; 1.75)	0.854	1.22 (−0.77; 3.2)	0.230	0.1 (−1.46; 1.66)	0.899	1.25 (−0.66; 3.17)	0.200	0.1 (−1.46; 1.66)	0.899	1.24 (−0.68; 3.17)	0.205
Marital status (Ref: Currently married/cohabiting)												
Previously married/cohabiting/partner deceased	0.26 (−2.01; 2.53)	0.822	−0.54 (−1; −0.08)	**0.020**	0.04 (−2.17; 2.25)	0.972	−0.38 (−0.82; 0.07)	0.102	0.03 (−2.17; 2.24)	0.975	−0.35 (−0.8; 0.1)	0.128
Never married/cohabiting	−2.42 (−4.16; −0.69)	**0.006**	−4.45 (−10.67; 1.78)	0.162	−1.92 (−3.62; −0.23)	**0.026**	−4.81 (−10.82; 1.21)	0.117	−1.93 (−3.62; −0.23)	**0.026**	−4.78 (−10.82; 1.27)	0.121
Education level (Ref: No formal education)												
Primary	−0.01 (−0.56; 0.55)	0.984	1.74 (0.92; 2.56)	**< 001**	0.01 (−0.53; 0.55)	0.970	1.26 (0.46; 2.06)	**0.002**	0.01 (−0.53; 0.55)	0.975	1.25 (0.45; 2.06)	**0.002**
Secondary	1.2 (0.38; 2.02)	**0.004**	7.42 (5.42; 9.42)	**< 001**	1.17 (0.34; 1.99)	**0.006**	6.56 (4.6; 8.52)	**< 001**	1.16 (0.34; 1.99)	**0.006**	6.56 (4.59; 8.53)	**< 001**
Tertiary	2.28 (0.52; 4.04)	**0.011**	7.74 (−0.84; 16.33)	0.077	2.08 (0.32; 3.85)	**0.021**	7.82 (−0.46; 16.11)	0.064	2.08 (0.31; 3.84)	**0.021**	8.14 (−0.17; 16.45)	0.055
Household asset status (Ref: Quintile = 1)												
2	0.32 (−0.39; 1.04)	0.372	−0.06 (−0.67; 0.55)	0.840	0.33 (−0.36; 1.02)	0.351	−0.13 (−0.73; 0.48)	0.675	0.33 (−0.36; 1.03)	0.350	−0.18 (−0.79; 0.43)	0.568
3	0.66 (−0.07; 1.4)	0.076	0.03 (−0.57; 0.62)	0.934	0.7 (−0.03; 1.43)	0.059	−0.06 (−0.67; 0.54)	0.834	0.71 (−0.02; 1.43)	0.058	−0.12 (−0.74; 0.5)	0.702
4	0.85 (0.11; 1.6)	**0.025**	0.28 (−0.32; 0.88)	0.367	0.78 (0.03; 1.53)	**0.042**	−0.01 (−0.62; 0.61)	0.986	0.78 (0.03; 1.53)	**0.041**	−0.06 (−0.68; 0.57)	0.854
5	2.84 (2.13; 3.55)	**< 001**	0.82 (0.23; 1.41)	**0.007**	2.45 (1.72; 3.18)	**< 001**	0.36 (−0.27; 0.98)	0.261	2.45 (1.72; 3.19)	**< 001**	0.3 (−0.33; 0.93)	0.344
Consumption alcohol status (Ref: Never consumption)												
Current non problematic					−0.23 (−0.73; 0.26)	0.355	0.2 (−0.25; 0.64)	0.385	−0.23 (−0.73; 0.26)	0.354	0.21 (−0.23; 0.66)	0.344
Current problematic					−0.89 (−1.54; −0.24)	**0.007**	−0.05 (−0.76; 0.65)	0.882	−0.89 (−1.54; −0.24)	**0.007**	−0.03 (−0.74; 0.68)	0.939
Ever consumption					−0.63 (−1.5; 0.24)	0.157	0.6 (−0.03; 1.22)	0.060	−0.63 (−1.5; 0.25)	0.160	0.6 (−0.03; 1.22)	0.062
Smoking status (Ref: Never smoked)												
Current smoker					−2 (−2.59; −1.41)	**< 001**			−2 (−2.59; −1.41)	**< 001**	−2.3 (−6.35; 1.75)	0.266
Former smoker					−0.12 (−0.75; 0.5)	0.706	−2.35 (−6.4; 1.7)	0.255	−0.11 (−0.74; 0.52)	0.724		
Chewing tobacco use (Ref: No)												
Yes					−0.72 (−1.54; 0.11)	0.091	−0.79 (−1.2; −0.37)	**< 001**	−0.72 (−1.55; 0.11)	0.090	−0.8 (−1.22; −0.39)	**< 001**
Servings bread/day					−0.02 (−0.12; 0.08)	0.721	0.42 (0.27; 0.57)	**< 001**	−0.02 (−0.12; 0.08)	0.719	0.42 (0.27; 0.56)	**< 001**
Fruit serving/day					0.06 (−0.05; 0.17)	0.281	0.07 (−0.04; 0.17)	0.196	0.06 (−0.05; 0.17)	0.274	0.07 (−0.04; 0.18)	0.206
Vegetable serving/day					0.11 (0.03; 0.2)	**0.008**	0.06 (−0.01; 0.13)	0.106	0.11 (0.03; 0.2)	**0.008**	0.06 (−0.01; 0.13)	0.105
Physical activity					0 (0; 0)	0.622	0 (0; 0)	0.079	0 (0; 0)	0.618	0 (0; 0)	**0.046**
Eating out/week					0.06 (−0.03; 0.16)	0.209	0.19 (−0.05; 0.43)	0.125	0.06 (−0.03; 0.16)	0.207	0.19 (−0.05; 0.42)	0.130
Sugar sweetened beverage/week					0.13 (−0.02; 0.29)	0.086	0.47 (−0.17; 1.11)	0.150	0.13 (−0.02; 0.29)	0.087	0.48 (−0.17; 1.12)	0.147
Sleep (hours/night)					−0.06 (−0.24; 0.11)	0.483	−0.15 (−0.31; 0.01)	0.062	−0.06 (−0.24; 0.11)	0.487	−0.15 (−0.31; 0.01)	0.060
Sitting (hours/day)					0 (0; 0)	0.907	0 (0; 0)	**0.013**	0 (0; 0)	0.907	0 (0; 0)	**0.011**
TB status (Ref: No)												
Yes									−0.19 (−1.64; 1.26)	0.798	−0.75 (−2.58; 1.08)	0.422
Don’t Know									-	-	−0.32 (−2.52; 1.87)	0.772
Not answered									-	-	−1.82 (−7.54; 3.9)	0.533
Menopause (Ref: Pre-menopause)												
Peri-menopause									-	-	0.35 (−0.27; 0.98)	0.269
Post-menopause									-	-	−0.11 (−0.54; 0.32)	0.627
Number of pregnancies											0.06 (−0.02; 0.14)	0.153
	Model 1: R^2^ Male: 0.1855; R^2^ Female: 0.1022				Model 2: R^2^ Male: 0.2502; R^2^ Female: 0.1758				Model 3: R^2^ Male: 0.2638; R^2^ Female: 0.1642			

Model 1: Association between sociodemographic variables (age, ethnicity, education level, marital status and SES (quintiles)) and BMI. We included all variables with p < 0.20 from the bivariate analyses.

Model 2: Lifestyle (behavioural) variables: alcohol consumption, tobacco exposure, MVPA, sedentary behaviour). Include all variables with p < 0.20

Model 3: Model 1 and Model 2, in addition with TB status, menopause, and number of pregnancies

CI 95%: Confidence Interval 95%

#### Structural equation model (SEM)

**Figure 1.**
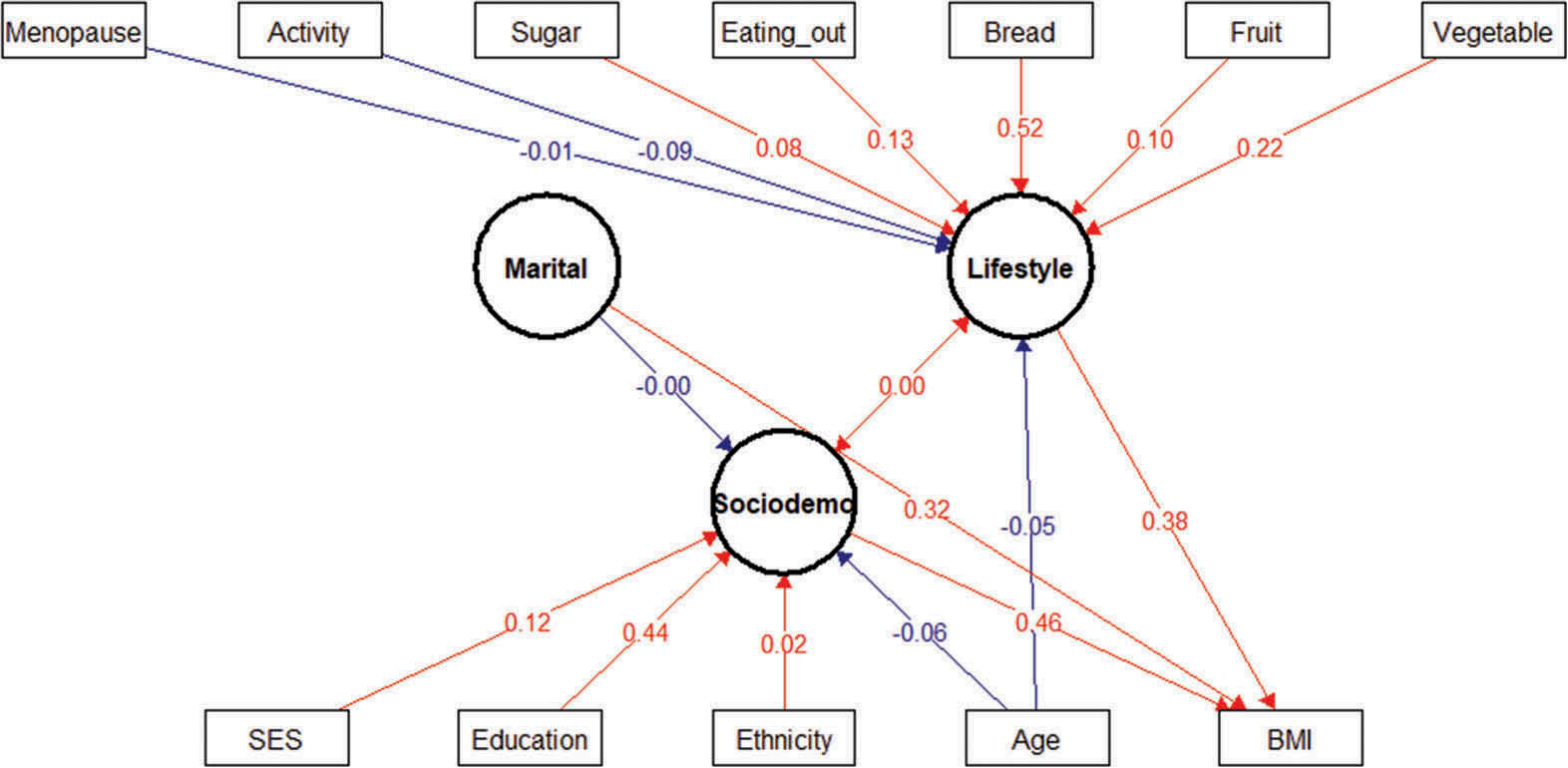
Structural equation model (SEM) of interaction between sociodemographic and lifestyle factors and BMI in women. Latent variables are drawn as circles. Manifest or measured variables are shown as squares. Numbers refer to interactions (correlations) between an indicator and its factor. The blue lines indicate negative interactions and the in red lines indicate positive interactions. All interactions represented in the figure are significant. The variables were tested as both latent and manifest variables and only the most significant interactions were reported in the study.

**Figure 2.**
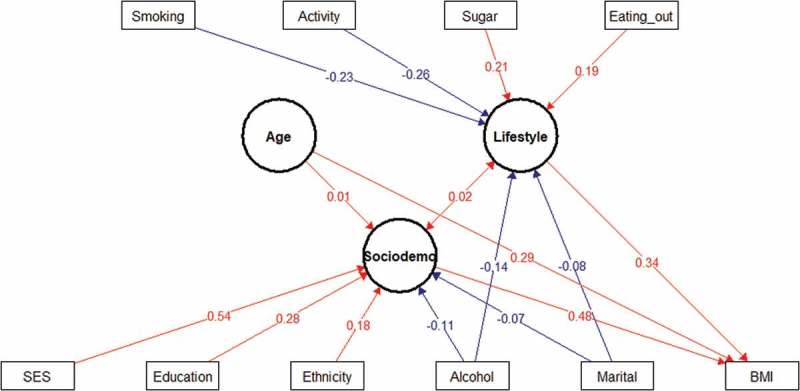
Structural equation model (SEM) of interaction between sociodemographic and lifestyle factors and BMI in men. Latent variables are drawn as circles. Manifest or measured variables are shown as squares. Numbers refer to interaction (correlation) between an indicator and its factor. The blue lines indicate negative interactions and the in red lines indicate positive interactions. All interactions represented in the figure are significant. The variables were tested as both latent and manifest variable and only the most significant interactions were reported in the study.

The SEM results showed the differences in determinants of BMI between men and women. For women (Figure 1), being a widow (marital status) was found to be associated with a decrease in BMI, and menopausal status had an indirect effect on BMI through lifestyle changes. In women, the direct effect of SES on BMI is low, but the indirect effect is considerable since we found that SES influenced BMI through increased eating out, bread consumption, sugar sweetened beverage, fruit and vegetable intake being associated with an increase in BMI. The SEM confirmed that men who never married, but who smoked and consumed alcohol had lower BMI (Figure 2). Unlike the hierarchical model, physical activity was associated with lower BMI in the SEM. Ethnicity, sugar-sweetened beverages and eating out are latent factors revealed by SEM to be contributing to an increase of BMI.

## Discussion

This cross-sectional study describes the BMI distribution in rural Burkina Faso in the 40–60-year-old age group. The results showed that there was a difference in BMI between men and women and although there was a low prevalence of overweight and obesity, men were more likely to be overweight and obese than women. The present results differ from those in previous studies that reported higher BMI in adult women than men in Burkina Faso and other sub-Saharan African countries (rural and urban). In the STEPwise approach to Surveillance (STEPS), national survey for non-communicable diseases, it was reported that women were more overweight and obese than men [17]. Other studies in urban Burkina Faso or hospital (outpatients) settings reported that women were more overweight and obese than men [12,22–25]. In their study in four sub-Saharan countries (Nigeria, South Africa, Tanzania, Uganda) Ajayi et al. also reported the gender difference in favour of women [11]. Nonetheless, the overall prevalence of overweight and obesity in our study is similar to those reported in the STEPS surveys for rural Burkina Faso (overweight: 8.5% vs 9.4% and obesity: 1.8% vs 1.9%). Our study revealed a high prevalence of underweight among men and women. We attempt to explain our findings from the perspective of the Developmental Origins of Health and Disease paradigm (DOHaD) which is a multidisciplinary field that examines how environmental factors acting during the phase of developmental plasticity impact organisms in later life. Since Barker proposed this model by showing that low birth weight is associated with an increased risk of CVD later in life, this concept has been extensively studied [26]. Some of the hypotheses are that foetal and perinatal events, such as maternal over-and-undernutrition, were central to determining the risk of developing chronic metabolic disorders, and events such as early life exposure to famine during the high plasticity periods (e.g. under five years or during puberty) could result in particular conditions later in life [27,28]. We considered that Burkina Faso experienced two major famine episodes in 1974 and in 1985 and estimated the proportion of our study population who would have been exposed to famine as young children or when going through puberty (foetal-to-under-five years old (1 to 5 years) or pubertal age (12–15 years)). We found that about half of our study population could have suffered the burden of these famines, and this led us to check for particular patterns of underweight according to age, there were however no significant differences. We then examined the results from previous studies and found that women (15–49 years of age) in Burkina Faso tend to have a high prevalence of underweight. The 2003 DHS study reported 16.0% of women to be underweight, the 2008 Nutrition and Food Security Survey reported 19.7%; and the DHS study of 2010 reported 19.0% (16,27,28).

### Factors associated with BMI

Among different factors, older age was found to be associated with a decrease in BMI in men but not in women in the hierarchical model (Table 3). This association was direct in men as shown in the SEM, but negative and indirect (acting through lifestyle and sociodemographic factors) in women (Figures 1 & 2). These results differ from Sagna and collaborators who concluded in their study in peripheral and central urban areas in Burkina Faso that increased obesity was associated with older age [22].

Marital status was found to be associated with BMI. Men who never married/cohabited were found to have a lower BMI than those who were married. This may be explained by two reasons: the first is that they may have lower food intake since in the rural culture, the female partner is responsible for food preparation; the second may be the psychological burden of being single since in this community being a single man at that age is subject to stigmatization.

Education level was found to be associated with BMI. In men, secondary and tertiary levels were associated with higher BMI while in women only primary and secondary education were associated (even though the number of individuals in the secondary and tertiary level groups were small) with BMI. It is important to note that women with secondary school education had a BMI level that was higher than those with no formal education (7.42 95% CI: 5.42; 9.42). This trend has been reported in previous studies [23,25]. Furthermore, the SEM results demonstrate that education is the most important driver of BMI for women (Figure 1) with small indirect effects via SES [23,25].

The SES of the population was not associated with BMI in women. The association in men was found only at the highest quintile (Q5) (2.46, 95% CI: 1.73; 3.19). This difference could be due to gender inequalities. Thus, women from wealthier households did not have higher BMI levels.

Problematic alcohol consumption was associated with a decrease of BMI in men (−0.88, 95% CI: −1.52; −0.23), but not in women. Similar associations have been reported in other populations [29,30]. The association between alcohol and BMI had long been controversial. Some studies show a positive relationship of alcohol intake on BMI and others show a negative relationship, with most of these studies using the amount of alcohol consumed as an independent variable. In our study, we did not use this approach, preferring the assessment of drinking problems using the CAGE method. The relationship between alcohol and BMI in our population showed a direct effect on BMI and this was confirmed through the SEM method [29,30].

It was found that tobacco exposure was associated with lower BMI in men (−2.05, 95% CI: −2.63; −1.47) as well as women (−0.76, 95% CI: −1.17; −0.35). Men were found to use mainly smoking tobacco whilst women were consuming predominantly chewing tobacco [31–34]. These associations are in line with the literature for studies in many countries, which demonstrate that smokers have up to a 5 kg lower weight than non-smokers [35,36]. In this study, the difference in BMI between smokers and non-smokers was about 2 kg/m^2^ corresponding to approximatively a 6 kg difference in weight for a man with a height of 1.7 m. This can be physiologically explained by the fact that nicotine increases thermogenesis in adipose tissue, partly by increasing lipolysis and subsequent recycling of fatty acids into triglycerides [37]. Smoking increases 24-h energy expenditure by ~ 10% and increases energy expenditure more during exercise and after eating than while at rest [38]. A 10% increase in metabolic rate, corresponding to an expenditure of 200 kcal per 24 h, seems small; however, assuming that there is no change in caloric intake, this increase in energy expenditure caused by nicotine can result in the loss of 10 kg in body weight over 1 year [39].

The negative association between smoking and BMI was also observed using the SEM, showing the direct effect on BMI in men (Figure 2). The lower weight decrease among women compared to men may be due to differences in nicotine absorption through the digestive tract [40].

Two dietary factors were found to be associated with BMI. The consumption of bread was positively associated with BMI in women (0.42, 95% CI: 0.27; 0.56) and the consumption of vegetables in men (0.11, 95% CI: 0.03; 0.2). Considering the low median BMI of our population, this could be seen more as an indicator of a diverse diet rather than as a risk factor of obesity. The SEM showed that bread and vegetable consumption were directly related to BMI in women, while eating out and sugar consumption were directly related to higher BMI in men (Figure 2).

## Conclusion

This study provides prevalence data on obesity categories and on factors that may influence obesity. It gives insights into the modulators of BMI in a rural community in Burkina Faso. The results were generated from a cross-sectional population study and cannot be considered as causal factors for BMI, and therefore there is a need for longitudinal data to confirm the model. Nonetheless the findings of this study show the distribution of BMI among middle-aged adults in rural Burkina Faso and highlight that being underweight is far more prevalent than being overweight/obese. It was interesting to observe a strong correlation of both SES and education level with BMI despite the low prevalence of formal education and high quintile SES categories in this population. This suggests that improvements in education levels or household SES may be paralleled by an increase in BMI. Thus, attempts to improve the sociodemographic profile within this community must go hand-in-hand with education programs on healthy eating and the health-related dangers of obesity. This study also raised concerns regarding the high prevalence of other cardiometabolic risk factors, such as tobacco use and alcohol intake. Finally investigating other indices of body composition, such as waits circumference and visceral fat, could be more relevant to assessing cardiometabolic risk factors than BMI.

### Strengths and limitations

This study in a rural Burkina Faso allowed us to pinpoint factors associated with BMI. Although reporting low prevalence of overweight/obesity, these results pertain to rural Burkina Faso and do not represent the situation in urban communities in Burkina Faso. Despite our efforts to provide accurate measurements, limitations of the study were present. For example, bread is not a good indicator of carbohydrate intake in Burkina Faso as bread is not a part of the usual eating habits as it is rarely part of meals, self-reported menopausal status is less accurate that an in-depth analysis, and fruit and vegetable consumption may be inaccurate as the levels were very low. An accurate dietary assessment would have provided a better understanding of several factors associated with BMI.

## Paper context

Sub-Saharan Africa is displays regional and sex-specific disparities in obesity. This population-based cross-sectional study implemented in the Nanoro’s HDSS area showed that there is a high prevalence of underweight among middle-aged adults with women suffering the highest burden. We found that SES, education, tobacco (smoking and chewing) and alcohol consumption are correlated with BMI in the rural Burkina Faso.
